# Compliance with the World Health Organization’s surgical safety checklist and related postoperative outcomes: a nationwide survey among 172 health facilities in Ethiopia

**DOI:** 10.1186/s13037-022-00329-6

**Published:** 2022-06-10

**Authors:** Manuel Kassaye Sibhatu, Desalegn Bekele Taye, Senedu Bekele Gebreegziabher, Edlawit Mesfin, Hassen Mohammed Bashir, John Varallo

**Affiliations:** 1 Jhpiego Ethiopia, Johns Hopkins University Affiliate, Mailbox 607. Bole subcity, Woreda 13, House No. B17/3, Addis Ababa, Ethiopia; 2 Ministry of Health of Ethiopia, Addis Ababa, Ethiopia; 3 Armauer Hansen Research Institute, Addis Ababa, Ethiopia; 4 Jhpiego Corporation, Johns Hopkins University Affiliate, Baltimore, USA

**Keywords:** Surgical safety checklist, Completeness, Compliance, Surgical complication, Adverse event, Cross-sectional, Ethiopia

## Abstract

**Background:**

Ministry of Health (MOH) of Ethiopia adopted World Health Organization’s evidence-proven surgical safety checklist (SSC) to reduce the occurrence of surgical complications, i.e., death, disability and prolong hospitalization. MOH commissioned this evaluation to learn about SSC completeness and compliance, and its effect on magnitude of surgical complications.

**Methods:**

Health institution-based cross-sectional study with retrospective surgical chart audit was used to evaluate SSC utilization in 172 public and private health facilities in Ethiopia, December 2020–May 2021. A total of 1720 major emergency and elective surgeries in 172 (140 public and 32 private) facilities were recruited for chart review by an experienced team of surgical clinicians. A pre-tested tool was used to abstract data from patient charts and national database. Analyzed descriptive, univariable and bivariable data using Stata version-15 statistical software.

**Results:**

In 172 public and private health facilities across Ethiopia, 1603 of 1720 (93.2%) patient charts were audited; representations of public and private facilities were 81.4% (*n* = 140) and 18.6% (*n* = 32), respectively. Of surgeries that utilized SSC (67.6%, 1083 of 1603), the proportion of SSC that were filled completely and correctly were 60.8% (659 of 1083). Surgeries compliant to SSC guide achieved a statistically significant reduction in perioperative mortality (*P* = 0.002) and anesthesia adverse events (*P* = 0.005), but not in Surgical Site Infection (*P* = 0.086). Non-compliant surgeries neither utilized SSC nor completed the SSC correctly, 58.9% (944 of 1603).

**Conclusions:**

Surgeries that adhered to the SSC achieved a statistically significant reduction in perioperative complications, including mortality. Disappointingly, a significant number of surgeries (58.9%) failed to adhere to SSC, a missed opportunity for reducing complications.

**Supplementary Information:**

The online version contains supplementary material available at 10.1186/s13037-022-00329-6.

## Introduction

Globally, over 200 million surgeries are performed annually [1]. However, the growing number of surgical complications remain a public concern [2]. In Africa, the magnitude of perioperative mortality rate is high, 3.4–7% [3, 4]. Anderson et al. (2013) showed that adverse events occurred in 14.4% (IQR,12.5–20.1%) of 16,424 surgeries, 7.3% were surgical site infection [5]. In Ethiopia, a pooled prevalence of SSI estimated in a meta-analysis that involved 13,136 surgeries was 12.3% (95%CI:10.19–14.42) [6].

To ensure safety, Ministry of Health (MOH) of Ethiopia integrated the World Health Organization’s surgical safety checklist (SSC) into its surgical care strategy [7, 8]. White et al. (2021) showed the benefits of SSC in reducing surgical complications; mortality by 23% (RR 0.77; 95%CI 0.67–0.89) and risk of infections by 53% (RR 0.47; 95%CI 0.40–0.55) [9]. Another longitudinal study of SSC impact on clinical endpoints in 3733 surgeries found a significant reduction in perioperative death from baseline of 1.5 to 0.8% (*P* = 0.003), and other complications from baseline 11.0 to 7.0% (*P* < 0.001) [10, 11].

However, local evidence on SSC utilization and impact on surgical complications is lacking [12]. Hence, MOH commissioned this evaluation to generate evidence to shape Ethiopia’s surgical and anesthesia care program.

## Methods

In 2016, MOH integrated SSC strategies into national surgical care program, and tracked SSC utilization through a district health information software-2 (DHIS2) in public facilities.

An institution-based cross-sectional study was used to retrospectively evaluate SSC utilization by reviewing surgical charts in 172 public and private health facilities in Ethiopia, December 2020–May 2021. Evaluation facilities were enrolled using a multi-stage stratified random sampling technique. A single population proportion formula was used to determine the minimum sample size; (*n* = z^2^pq/e^2^/1 + (z^2^pq/e^2^)*N) for a finite population with a 0.5 population proportions, 5% margin of error and 95% level of confidence. Of the total study sample of 203 (163 public and 40 private) facilities, 163 public surgical care facilities were selected from MOH’s health facilities registry (26 tertiary care specialized referral hospitals, 75 secondary care general hospitals, and 181 primary care facilities). The sample size for each stratum (level of care) was distributed using a proportion-to-size allocation method, size being the number of facilities in a specific stratum, i.e. specialized hospitals (n_r_ = 15), general hospitals (n_g_ = 43) and primary facilities (n_p_ = 105). Similarly, 88.9% (40 out of 45) private surgical care facilities were enrolled.

At facility-level, ten random charts were selected from surgical logbooks*.* All major surgeries performed within 90 days of evaluation were included while those performed beyond 90 days and minor surgeries were excluded. A total of 1720 surgical patients’ charts were audited by an experienced team of surgical clinician. A pre-tested data abstraction tool was used to collect information on SSC completeness, compliance, surgical complications (perioperative mortality, SSI, and anesthesia adverse event), and SSC completeness rate self-reported by facilities.

### Statistical analysis

Data were cleaned on weekly basis, checked for completeness, correctness and consistency, and finally entered into a Redcap study database. Data exported to Stata version-15 software for computing descriptive statistics and compare categorical variables by chi-square test. Statistically significance declared at *P* < 0.05.

### Ethical consideration

Ethical clearance was secured from the Armauer Hansen Research Institute (AHRI). MOH issued a letter of support to health bureaus and facilities.

### Operational definition

#### Major surgery

An institution-based invasive intervention involving the incision, excision, manipulation, or suturing of tissue, usually requiring regional or general anesthesia or sedation.

#### Surgical safety checklist completeness

Assesses whether all entries of the checklist are completed and filled correctly as indicated in the SSC guide. *Numerator*: Count of surgical procedures that utilized SSC, and filled completely and correctly. *Denominator*: Total number of major surgeries performed within 90 days of chart audit.

#### Non-compliance rate

Assesses deviations from surgical safety guide. *Numerator:* Count of major surgeries that didn’t utilize the checklist and surgeries whose SSC was neither complete nor filled correctly. *Denominator:* Total number of major surgeries performed within 90 days of chart audit.

## Results

**Fig. 1:**
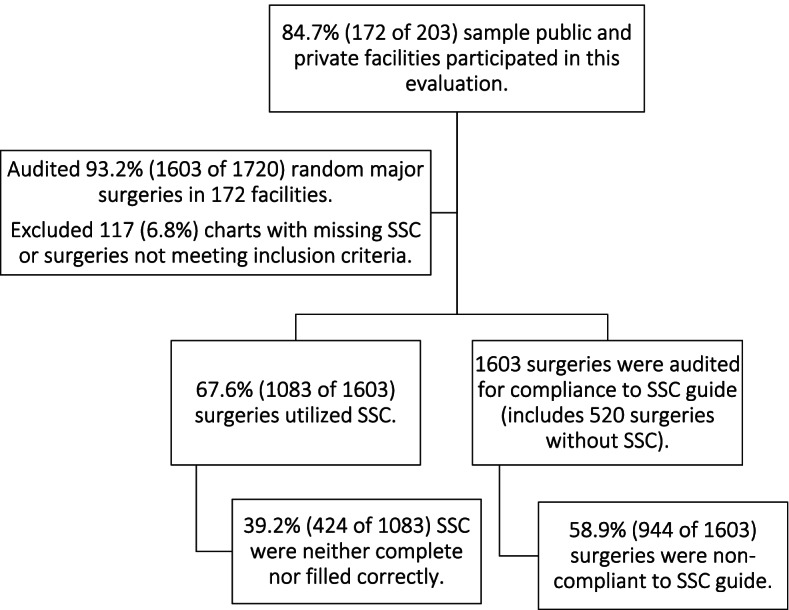
Surgical Safety Checklist utilization during emergency and elective surgeries in public and private health facilities in Ethiopia, December 2020 to May 2021

Out of the sample 203 health facilities, 172 (84.7% response rate) participated in this evaluation; public and private health facilities accounted for 81.4% (*n* = 140) and 18.6% (*n* = 32) of the evaluation facilities, respectively. Of the total surgical patient charts audited, 93.2% (1603 of 1720), the proportion of surgeries that utilized SSC were 67.6% (1083 of 1603) (Fig. 1). SSC utilization rate was highest in public specialized hospitals (85%) and lowest in private health facilities (23.1%). An aggregate SSC completeness rate was 60.8% (659 of 1083) while the remaining (39.2%) were neither complete nor filled correctly (Table 1). SSC completeness rate self-reported to the DHIS2 by facilities was 81% while completeness rate computed by retrospective chart audit was 60.8%.

**Table 1:** Surgical safety checklist completeness during an emergency and elective surgeries in public and private health facilities in Ethiopia, December 2020 to May 2021

Facilities evaluated by level of Health care		Number of surgical patient chart		Surgeries that utilized SSC		Completeness or correctness of SSC	
Level of care^a^	Facilities. N(%)	Chart selected	Charts reviewed	Yes, N (%)	No, N (%)	Yes, N (%)	No, N (%)
Public Specialized Hospitals	16 (9.3%)	160	160 (100%)	136 (85%)	24 (15%)	80 (58.8%)	56 (41.2%)
Public General Hospitals	38 (22.1%)	380	370 (97.4%)	275 (74.3%)	95 (25.7%)	159 (57.8%)	116 (42.2%)
Public Primary Hospitals	86 (50%)	860	800 (93%)	609 (76.1%)	191 (23.9%)	382 (62.7%)	227 (37.3%)
Private Hospitals	32 (18.6%)	320	273 (85.3%)	63 (23.1%)	210 (76.9%)	38 (60.3%)	25 (39.7%)
Total	172 (100%)	1720	1603 (93.2%)	1083 (67.6%)	520 (32.4%)	659 (60.8%)	424 (39.2%)
1720 (100%)			1603 (100%)		1083 (100%)		

^a^Specialized hospital: tertiary level of care that serves 3.5 to 5.0 million people. General hospital: secondary level of care that serves 1 to 1.5 million people. Primary hospital: primary level of care that serves 60,000–100,000 people with an average inpatient capacity of 35 beds and has direct referral linkage with nearby primary care units (health centers and health posts). Health center (a.k.a. operation room blocks): primary level of care facility that typically has the capacity for providing emergency obstetric delivery services, and furnished by Ministry of Health to provide additional emergency and essential surgical care (primarily the Bellwether surgical procedures, i.e., Cesarean section, laparotomy, and open fracture management. (Ministry of Health. Health Sector Transformation Plan (2015/16–2019/20). Federal Democratic Republic of Ethiopia Ministry of Health. Addis Ababa; 2015)

The proportion of surgeries that neither complete nor filled the SSC correctly were 58.9% (944 of 1603). Non-compliance to SSC was most prevalent in public primary and private hospitals, 23.6 and 14.7%, respectively (Table 2). SSC adherence was achieved a significantly reduction in perioperative mortality (*P* = 0.002), and anesthesia adverse events (*P* = 0.005).

**Table 2:** Non-compliance to surgical safety checklist guide during major emergency and elective surgeries in public and private health facilities in Ethiopia, December 2020 to May 2021

Level of Health care	Number of surgical charts reviewed N(%)	Surgeries non-compliant to SSC guide^a^		
		Didn’t utilize SSC. N(%)	Incomplete or incorrect SSC	Non-compliant, total
Public Specialized Hospitals	160 (100%)	24 (15%)	56 (41.2%)	80 (50.0%)
Public General Hospitals	370 (97.4%)	95 (25.7%)	116 (42.2%)	211 (13.2%)
Public Primary Hospitals	800 (93%)	191 (23.9%)	227 (37.3%)	418 (26.1%)
Private Hospitals	273 (85.3%)	210 (76.9%)	25 (39.7%)	235 (14.7%)
Total	1603 (93.2%)	520 (32.4% of 1603)	424 (39.2% of 1083)	944 (58.9%)

^a^Note the difference between the denominators for ‘Didn’t utilize SSC’ (32.4% of 1603 charts reviewed) and the denominator for ‘Incomplete or incorrect SSC’ (39.2% of 1083 SSC used)

Though the reasons for non-compliance varied by level of care, the most frequently mentioned reasons were lack of knowledge, shortage of time, and staff unwillingness. Staff unwillingness was the commonest reason in public health care units (41%) while shortage of time was the commonest among public specialized hospitals, 46.1% (Table 3).

**Table 3:** Reasons for non-compliant use of surgical safety checklist in public and private health facilities in Ethiopia in a 90-day interval of the study period, December 2020 to May 2021

Reasons for non-compliance^a^	Level of health care facilities			
	Public Specialized Hospitals	Public Generalized Hospitals	Public Primary Hospitals	Private Hospitals
Shortage of time	6 (46.1%)	9 (31.0%)	9 (14.7%)	4 (14.3%)
Staff unwillingness	2 (15.4%)	2 (6.9%)	25 (41%)	7 (25%)
Lack of knowledge	3 (23.1%)	5 (17.2%)	13 (21.3%)	12 (42.9%)
Resource shortage	0	10 (34.5%)	9 (14.7%)	3 (10.7%)
Other	2 (15.4%)	3 (10.3%)	5 (8.2%)	2 (7.1%)

^a^Multiple reasons options were allowed

Adherence to SSC was associated with a statistically significantly reduction in perioperative mortality (*P* = 0.002), and anesthesia adverse events (*P* = 0.005). However, effect on SSI was not statistically significant (*P* = 0.086) (Tables 4, 5 and 6).

**Table 4:** Association between surgical safety checklist utilization and occurrence of surgical site infections in public and private health facilities in Ethiopia, December 2020 to May 2021

Key Performance Indicator (KPI)	Surgical Safety Checklist Utilization			
		yes	no	*P* value
Surgical site infections	Yes	45 (27.3%)	15 (9.1%)	p = 0.086
	No	90 (54.6%)	15 (9.1%)

**Table 5:** Association between surgical safety checklist utilization and occurrence of perioperative mortality in public and private health facilities in Ethiopia, December 2020 to May 2021

Key Performance Indicator (KPI)	Surgical Safety Checklist Utilization			
		yes	no	*P* value
**Death (perioperative mortality)**	Yes	23 (13.9%)	13 (7.9%)	p = 0.002
	No	112 (67.9%)	17 (10.3%)

**Table 6:** Association between surgical safety checklist utilization and occurrence of Anesthesia Adverse Events in public and private health facilities in Ethiopia, December 2020 to May 2021

Key Performance Indicator (KPI)	Surgical Safety Checklist Utilization			
		yes	no	*P* value
**Anesthesia adverse events**	Yes	17 (10.3%)	10 (6.1%)	p = 0.005
	No	118 (71.5%)	20 (12.1%)	

## Discussion

Surgeries that were compliant to SSC guide achieved a statistically significant reduction in surgical complication, primarily perioperative mortality (P = 0.002), and anesthesia adverse events (P = 0.005). Though over a third (67.6%) of the surgeries utilized safety checklists, only 659 (60.8%) checklists were filled completely and correctly. The non-compliance rate was unacceptably high, 58.9%.

The SSC completeness and compliance rate in Ethiopia was lower compared to findings in other studies. In Tanzania, Hellar et al. (2018) observed a higher SSC utilization rate, 68.8–99.4%; primary health care units (PHCU) performed significantly better (99.4%, *p* < 0.05) than higher-level hospitals [13]. An observational study by Schwendimann et al. (2019) evaluated adherence to SSC protocol in the University Hospital of Basel, Switzerland, and showed that the adherence rate for three domains of surgery ranged between 96 and 100% in timeout domain and sign-in domains, respectively, and 22% in sign-out. While the adherence rates for the timeout and sign-in was higher, the unavailability of key OR team members at sign-out time was the most common reason for low rate of completeness or adherence during the sign-out phase [14]. Though the SSC utilization rate in LMICs is lower, < 30%, compared to high-income countries (88–89%), in Ethiopia, SSC was integrated to national safe surgery strategy only recently [9]. In our evaluation, PHCUs scored higher SSC completeness rate (health centers, 67.1%; primary hospitals, 62.1%) compared to tertiary- and secondary care hospitals, 58.8% and 57.8%, respectively. This favors an argument that safety improvement in resource-constrained settings is feasible.

High non-compliance rate in private health facilities (14.7%) could be partly explained by the absence of surgical care programs and tracking of surgical performance metrics in private facilities. Private facilities could enhance the quality and safety of surgical care by adopting best safety practices [15]. Al-Qahtani et al. (2017) published experience of private hospitals who managed to increase SSC compliance rate (96.5%) through training and mentoring of staffs [16].

The discrepancy between SSC completeness rate in the national DHIS2 database (81%) and the retrospective audit (60.8%) may result from data quality issues or fidelity of the different assessment methods applied to compute completeness rates; compared to surveys, facility self-reports usually overestimate performance. Giles et al. (2016) depicted the impact of applying different measurement methods for estimating SSC completeness rate, 86% versus 27% through chart audit and direct observation, respectively [17].

The effect of SSC utilization on surgical complications was remarkable; those surgeries that adhered to SSC guide achieved a statistically significant reduction in surgical complications, perioperative mortality (*P* = 0.002) and anesthesia adverse events (*P* = 0.005), a finding that agrees with other evidences on clinical endpoints of adherent use of SSC [11].

The absence of statistically significant change in SSI rate (*P* = 0.086) could be attributed to several factors: weak SSI tracking systems, suboptimum adherence to infection prevention and control protocols, absence of post-discharge community SSI tracking, among other factors. The African Surgical Outcomes Study (ASOS) Group’s cohort of 11,422 surgeries in 25 African countries revealed an alarmingly high postoperative complications rates, 18·2% (95%CI 17·4–18·9]), and infection was the most common complication (10·2%) [18].

Interestingly, the most frequently mentioned reasons for non-compliance were surgical team’s lack of knowledge, unwillingness and shortage of time, and these reasons do vary by facility type; for instance, shortage of time was commonest in private and specialized hospitals, 36.8 and 50%, respectively. Though unwillingness and shortage of time was reported as one of the reasons for non-compliance, Tan et al. (2021) presented a favorable attitude towards SSC use from an inquiry of 846 surgical team in 138 hospitals in China; only 12.7% reported that the checklist ‘took a long time to complete’ and the majority (78.8%) reported that SSC was ‘easy to use’. In this study, OR staffs believed that SSC improved the surgical safety (90.4%), team communication (85.6%) and reduced errors (89.5%) [19]. Surgical team’s competency, attitude and communication on safety increases the likelihood of adherence and clinical outcomes [20–22]. Treadwell et al. (2014) described adherence and team communication as predictors of positive outcomes of SSC [23].

On the other hand, SSC could be adapted to standardize surgical practice among surgical team and minimize occurrence of surgical errors. Naqvi et al. (2022) showed that intraoperative practices of spinal surgeons in United Kingdom varied greatly, and 47.5% (29/61) of surgeons had been involved in wrong level spinal surgery [24].

One strength of this evaluation is that it is the first nation-wide assessment of surgical safety practices in public and private facilities in Ethiopia. Surgical safety practices were audited by an experienced team of clinicians who deeply understand the surgical care processes, and generated a quality data that could be generalizable to other surgical care settings. As this evaluation is owned by MOH, the evidence would be applicable to shape the national surgical program. Nonetheless, this evaluation didn’t use a prospective direct observation method to validate the use of SSC during live surgery, and the current retrospective evaluation may overestimate completeness and compliance to the use of checklist in real-time.

## Conclusions

Surgeries that adhered to the SSC guide achieved a statistically significant reduction in surgical complications, including perioperative mortality. While the aggregate adherence to SSC in public and private health facilities in Ethiopia was 60.8%, a significant number of surgeries (39.2%) missed an opportunity for reducing surgical complications. An overestimation of SSC utilization by self-report misleads decision-making.

Factors influencing compliance (lack of knowledge on how to complete safety checklist, team’s unwillingness and perceived shortage of time) need to be addressed through targeted training and coaching. Further research is warranted to better understand factors influencing adherence.

Leadership oversight and integration of SSC into existing perioperative risk assessment may increase likelihood of acceptance by surgical teams [9, 25, 26]. The evidence would inform Ethiopia’s safe surgery strategy.

## Supplementary Information

**Additional file 1.**

## Data Availability

The cleaned study data used to develop this manuscript is available at Ministry of Health and could be accessed through MOH and PI, a corresponding author, upon reasonable request.

## Abbreviations

AAE: Anesthesia adverse event
AHRI: Armauer Hansen Research Institute
ASOS: African Surgical Outcomes Study
DHIS2: District health information software-2
IRB: Institutional review board
MOH: Ministry of Health
POMR: Perioperative mortality rate
PHCU: Primary health care units
SSC: Surgical Safety Checklist
SSI: Surgical site infection
UBS: Unions Bank of Switzerland

## References

[1] Weiser TG, Regenbogen SE, Thompson KD, Haynes AB, Lipsitz SR, Berry WR (2008). An estimation of the global volume of surgery: a modelling strategy based on available data. Lancet.

[2] Kruk ME, Gage AD, Joseph NT, Danaei G, García-Saisó S, Salomon JA (2018). Mortality due to low-quality health systems in the universal health coverage era: a systematic analysis of amenable deaths in 137 countries. Lancet.

[3] Dandena F, Leulseged B, Suga Y, Teklewold B (2020). Magnitude and pattern of inpatient surgical mortality in Saint Paul’s hospital millennium medical college: Addis Ababa, Ethiopia: a three years’ retrospective review. Ethiop J Health Sci.

[4] Curcio D, Cane A, Fernández F, Correa J (2019). Surgical site infection in elective clean and clean-contaminated surgeries in developing countries. Int J Infect Dis.

[5] Anderson O, Davis R, Hanna GB, Vincent CA (2013). Surgical adverse events: a systematic review. Am J Surg.

[6] Shiferaw WS, Aynalem YA, Akalu TY, Petrucka PM (2020). Surgical site infection and its associated factors in Ethiopia: a systematic review and meta-analysis. BMC Surg.

[7] World Health Organization & WHO Patient Safety. Implementation manual: WHO surgical safety checklist. 1st ed: World Health Organization; 2008. https://apps.who.int/iris/handle/10665/70046.

[8] Ministry of Health (2016). National Surgical Care Strategic Plan: saving lives through safe surgery I (SaLTS I, 2016–2020). Addis Ababa, Ethiopia.

[9] White MC, Peven K, Clancy O, Okonkwo I, Bakolis I, Russ S (2021). Implementation strategies and the uptake of the World Health Organization surgical safety checklist in low- and middle-income countries: a systematic review and Meta-analysis. Ann Surg.

[10] Haynes AB, Weiser TG, Berry WR, Lipsitz SR, Breizat A-HS, Dellinger EP (2009). A surgical safety checklist to reduce morbidity and mortality in a global population. N Engl J Med.

[11] Haugen AS, Sevdalis N, Søfteland E (2019). Impact of the World Health Organization surgical safety checklist on patient safety. Anesthesiology..

[12] Tadesse H, Sibhatu M, Maina E, Bari S, Reynolds C, Richards K, Garringer K (2019). Saving lives through safe surgery (SaLTS) in Ethiopia: project implementation manual. Addis Ababa, Ethiopia.

[13] Hellar A, Tibyehabwa L, Ernest E, Varallo J, Betram MM, Fitzgerald L (2020). A team-based approach to introduce and sustain the use of the WHO surgical safety checklist in Tanzania. World J Surg.

[14] Schwendimann R, Blatter C, Lüthy M, Mohr G, Girard T, Batzer S (2019). Adherence to the WHO surgical safety checklist: an observational study in a Swiss academic center. Patient Saf Surg..

[15] Ramírez-Torres CA, Pedraz-Marcos A, Maciá-Soler ML, Rivera-Sanz F (2021). A scoping review of strategies used to implement the surgical safety checklist. AORN J.

[16] Al-Qahtani AS (2017). The surgical safety checklist: results of implementation in otorhinolaryngology. Oman Med J.

[17] Giles K, Munn Z, Aromataris E, Deakin A, Schultz T, Mandel C (2017). Use of surgical safety checklists in Australian operating theatres: an observational study. ANZ J Surg.

[18] Biccard BM, Madiba TE, Kluyts HL. African surgical outcomes study(ASOS) investigators: perioperative patient outcomes in the African surgical outcomes study: a 7-day prospective observational cohort study. Lancet. 2018;391. Available from. 10.1016/S0140-6736(18)30001-1.

[19] Tan J, Ngwayi JRM, Ding Z, Zhou Y, Li M, Chen Y (2021). Attitudes and compliance with the WHO surgical safety checklist: a survey among surgeons and operating room staff in 138 hospitals in China. Patient Saf Surg.

[20] McLaughlin N, Winograd D, Chung HR, Van de Wiele B, Martin NA (2014). Impact of the time-out process on safety attitude in a tertiary neurosurgical department. World Neurosurgery.

[21] dos Santos Diego LA, Salman FC, Silva JH, Brandão JC, de Oliveira Filho G, Carneiro AF (2016). Construction of a tool to measure perceptions about the use of the World Health Organization safe surgery checklist program. Brazilian J Anesthesiol (English Edition).

[22] Sorra J, Gray L, Streagle S (2016). AHRQ Hospital survey on patient safety culture: User’s guide. Agency for Healthcare Research and Quality.

[23] Treadwell JR, Lucas S, Tsou AY (2014). Surgical checklists: a systematic review of impacts and implementation. BMJ Qual Saf.

[24] Naqvi AZ, Magill H, Anjarwalla N (2022). Intraoperative practices to prevent wrong-level spine surgery: a survey among 105 spine surgeons in the United Kingdom. Patient Saf Surg..

[25] Sendlhofer G, Mosbacher N, Karina L, Kober B, Jantscher L, Berghold A (2015). Implementation of a Surgical Safety Checklist: Interventions to Optimize the Process and Hints to Increase Compliance. PLoS ONE.

[26] Wæhle HV, Haugen AS, Wiig S, Søfteland E, Sevdalis N, Harthug S (2020). How does the WHO surgical safety checklist fit with existing perioperative risk management strategies? An ethnographic study across surgical specialties. BMC Health Serv Res.

