# Supplementary material for: Compliance with the World Health Organization’s surgical safety checklist and related postoperative outcomes: a nationwide survey among 172 health facilities in Ethiopia
Source: Patient Saf Surg. 2022 Jun 10;16:20. doi: 10.1186/s13037-022-00329-6 (PMC9188150; doi:10.1186/s13037-022-00329-6)
Supplement: Supplementary file 1 — Additional file 1. [file 13037_2022_329_MOESM1_ESM.docx]

**ANNEX: DATA COLLECTION TOOLS_ Surgical Safety Checklist SSC_ Ethiopia**

**Tool 1:** **Data Abstraction Form for Chart and Register Review**

***Instruction****: Use this form to collect secondary data from surgical patient folder on* ***surgical safety checklist*** *(SSC). Please review for completeness and consistency of data and correct missing information or any errors as early as possible. Check all data sources for consistency and accuracy before handing over this form to your supervisors or investigators.*

***Process****: Use a random sampling technique to select patient charts. First identify eligible surgical cases from the surgical scheduling/operation room (OR) register/log book. For inaccessible or lost medical records, replace/substitute the chart as per the study procedures. For more information, please contact the site supervisor or investigators.* ***Thank you****.*

**SURGICAL SAFETY CHECKLIST (SSC) UTILIZATION**

*Here, please review 10 charts of* ***hospitalized patients*** *who underwent surgery in the past 90 days, or charts of surgical care patients who presented to ambulatory* ***surgical referral clinic*** *on* ***post-operation day****.*

***Appropriate use of checklist*** *is assessed determined by reviewing each domain (Sign-in, Time-out, Sign-out) in the Checklist. For instance, if an item was left incomplete, empty, unrecorded, or left blank, the use of the whole checklist will be labeled as “****incomplete/inappropriate****”.*

| Patient Chart | MRN | SSC code (assign #1-10) | Surgery used SSC, SSC attached to chart (Yes or No) | | SSC completed or filled correctly (Yes or No) | | Remark |
| --- | --- | --- | --- | --- | --- | --- | --- |
|  |  |  | Yes | No | Yes | No |  |
| Surgery 1 |  | SSC |  |  |  |  |  |
| Surgery 2 |  | SSC |  |  |  |  |  |
| Surgery 3 |  | SSC |  |  |  |  |  |
| Surgery 4 |  | SSC |  |  |  |  |  |
| Surgery 5 |  | SSC |  |  |  |  |  |
| Surgery 6 |  | SSC |  |  |  |  |  |
| Surgery 7 |  | SSC |  |  |  |  |  |
| Surgery 8 |  | SSC |  |  |  |  |  |
| Surgery 9 |  | SSC |  |  |  |  |  |
| Surgery 10 |  | SSC |  |  |  |  |  |
| Unit/facility score (out of 10 surgeries) | | | Utilized SSC: _____% |  | Completed SSC: ____% |  |  |
